# Remote multidisciplinary heart team meetings in immersive virtual reality: a first experience during the COVID-19 pandemic

**DOI:** 10.1136/bmjinnov-2021-000662

**Published:** 2021-03-05

**Authors:** Amir H Sadeghi, Ali R Wahadat, Adem Dereci, Ricardo P J Budde, Wilco Tanis, Jolien W Roos-Hesselink, Hanneke Takkenberg, Yannick J H J Taverne, Edris A F Mahtab, Ad J J C Bogers

**Affiliations:** 1 Department of Cardiothoracic Surgery, Erasmus Medical Center, Rotterdam, The Netherlands; 2 Department of Cardiology, Erasmus Medical Center, Rotterdam, The Netherlands; 3 Department of Cardiology, Haga Hospital, Den Haag, The Netherlands; 4 Department of Radiology and Nuclear Medicine, Erasmus Medical Center, Rotterdam, The Netherlands

**Keywords:** COVID-19, cardiology, health, cardiovascular diseases

**Summary box**

**What are the new findings?**

- Due to COVID-19 pandemic, gathering restrictions have challenged the organisation of physical multidisciplinary meetings, requiring innovative remote meeting methods, such as the immersive virtual reality (VR)-based method presented in this article.
- Immersive VR-based coronary revascularisation meetings were organised to enable remote multidisciplinary discussion between cardiac surgeons and cardiologists.

**How might it impact on healthcare in the future?**

- In the near future, extended reality platforms could overcome social distancing and gathering restrictions by enabling remote multidisciplinary collaboration for healthcare providers.
- VR-technology could have the potential to positively impact developments in pre-procedural medical planning, televirtuality and digital health solutions that could benefit both patients and physicians.

## Introduction

Virtual reality (VR) is an emerging technology that enables creation of digital objects and virtual animations in a digital immersive environment that can be visualised and interacted with through head mounted displays (HMD) and controllers.^1 2^ In the fields of cardiovascular medicine and surgery, an increasing number of reports have become available to demonstrate potential benefits of VR for education, surgical planning and simulation.^2–7^ In addition, VR has made its entrance into the world of communication and is an ongoing topic of interest in scientific research and promising new tools are being developed.^8^

Due to the recent COVID-19 outbreak, local authorities have implemented several protective measures such as physical distancing and gathering restrictions. These restrictions have also been partly implemented in hospitals, however, in the fields of cardiology and cardiac surgery, full restrictions could potentially be harmful to patients, and therefore, alternative meeting methods should be implemented locally. Recently, we have published an article in which we present examples of alternative methods for multidisciplinary meetings to minimise the risk of viral infection and to ensure good and ongoing regular patient care.^8^

By combining VR meeting platforms with various HMD’s, the user is able to immerse in a reality-like and fully 3D digital environment. VR-meeting platforms enable the users to get immersed in a virtual environment and provide them with digital tools (such as laser pointers and various meeting rooms) that enable VR-guided remote digital conferencing and televirtuality. By organising remote multidisciplinary meetings, direct physical interaction can be avoided, and the risk of viral transmission can be minimised. Until now, no studies on the organisation of clinical multidisciplinary heart team meetings in immersive VR are available in the literature. In order to study the feasibility, effectiveness and user experience of a VR-based multidisciplinary heart team meeting, we have set up an observational proof-of-concept study, which was accelerated by the COVID-19 pandemic. Here, we describe our first experience on the application of VR meeting platforms in the setting of multidisciplinary coronary revascularisation heart team meetings.

## Methods

### Experimental setup

**Figure 1:**
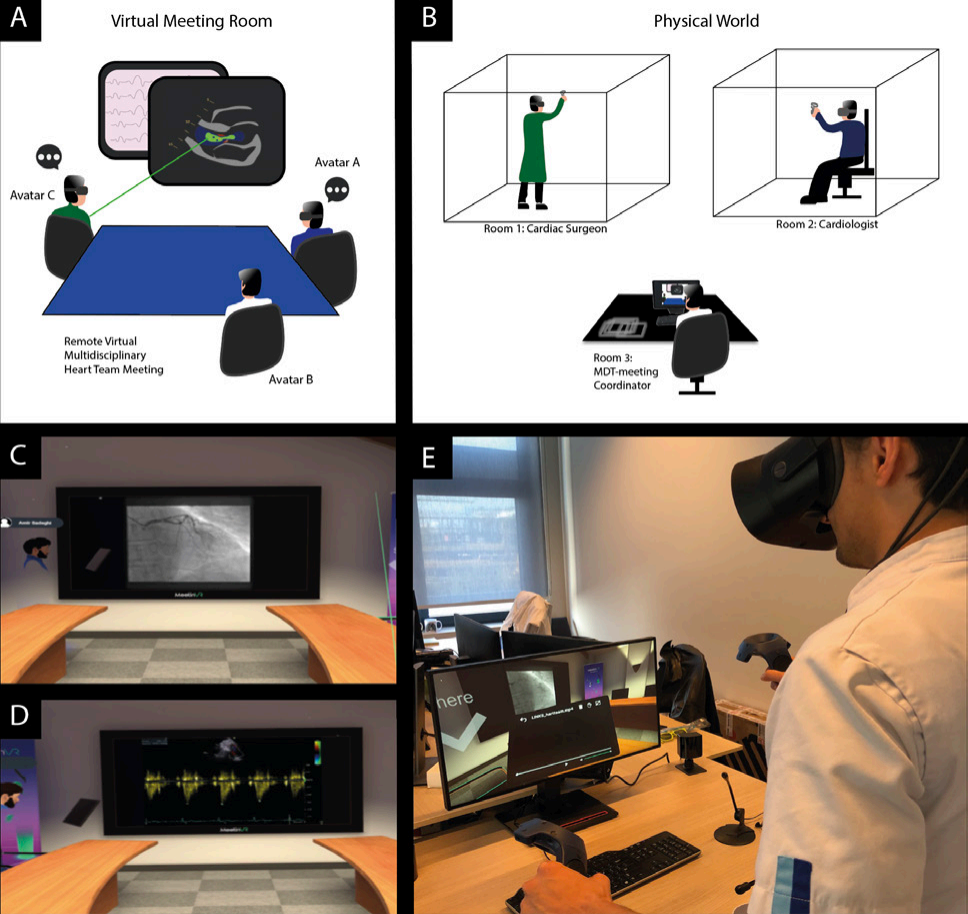
Immersive virtual reality based coronary revascularisation heart team meetings. (A, B) Illustrative depiction of the experimental setup for remote multidisciplinary heart team meetings. Participants in remote areas of the hospital (right panel) joined a virtual meeting room as an avatar (left panel) and discussed a clinical case of a patient with coronary artery disease that was presented by an MDT (multidisciplinary team meeting)-coordinator. (C, D) Several screenshots that have been acquired during a multidisciplinary remote and virtual reality-based heart team meeting. (E) In addition, photos present the hardware setup that enable remote virtual reality meetings.

At the Erasmus University Medical Center, a heart team meeting is held with at least an interventional cardiologist and a cardiothoracic surgeon. Five cardiothoracic surgeons (one in training) and five cardiologists were invited to participate in this study. Before the VR-meetings, all participants were briefed (5 min) on how to use the hardware and software. Immersive VR-based remote multidisciplinary coronary revascularisation heart team meetings were simulated according to local principles and with adherence to local gathering restriction rules (figure 1). Each VR-meeting consisted of at least two participants from both cardiology and cardiothoracic surgery department. A total of 10 meetings were organised consisting of at least one study participant (cardiologist/cardiac surgeon) and one resident cardiology/cardiothoracic surgery physician. Participants remotely joined a virtual room in a VR-based meeting platform (MeetinVR, Copenhagen, Denmark) by using VR-1 (Varjo, Helsinki, Finland) and Rift S (Oculus, Irvine, California, USA) HMD’s, VR-controllers, and high-performance Thinkstation (Lenovo, Quarry Bay, Hong Kong) computers. Experienced VR-users were on site to provide technical support during the meetings. During the VR-meetings, a coordinator (resident physician) provided heart team participants anonymised medical images (coronary angiography, echocardiogram, ECG and chest X-ray) of a patient with confirmed three-vessel coronary artery disease (history of hypertension, diabetes and good left ventricular function) who already had been discussed in an earlier heart team meeting.

### Objectives and questionnaires

The objective of this proof-of-concept study was to evaluate the feasibility and efficacy (being able to assess a case in VR) of organising remote VR-meetings to simulate heart team meetings. We defined feasibility as the ability to create a multidisciplinary meeting in VR to enable review of clinical imaging data remotely. Second, our aim was to study the subjective VR-experience and benefits of immersive meetings through questionnaires (online supplemental file S1) focused on: ease-of-use, immersiveness (engagement), usefulness and effectiveness, attitude toward (future) use,usefulness and effectiveness, and attitude toward (future) use. Questionnaires were created based on existing literature.^9–13^ A total of 25 questions were created and a Likert rating scale was used with items rated between 1 and 5 (online supplemental file S1). In addition, the final decision and recommendation of all virtual heart teams were documented and compared with the clinical recommendation of the physical meeting.

10.1136/bmjinnov-2021-000662.supp1 Supplementary data

### Data analysis

Data were analysed by using Excel 2020 V.16.43 (Microsoft, Redmond, Washington, DC, USA). Categorical discrete data (Likert rating scales) are represented as counts/proportions.

## Results

### Participants

Nine participants were men and one was female. Eight out of ten participants had at least 5 years of experience in physical heart team meetings on a weekly basis. Two had >3 years of experience for at least 1–2 times a month. Most study participants (n=6) did not have any VR experience before, 3 had basic VR experience and 1 uses VR on a regular basis. None of the participants did have any experience in immersive VR-based remote meetings.

### Feasibility

All VR-heart team meetings were organised successfully. All clinical imaging data were successfully visualised and assessed in VR (figure 1). In all meetings, the team suggested coronary artery bypass grafting as the most suitable therapy. This corresponded with the clinical recommendation. The duration of the meetings was comparable to regular physical meetings with a maximum of 10 min (excluding 5 min of briefing).

### Ease-of-use, immersiveness, usefulness, and effectiveness

**Figure 2:**
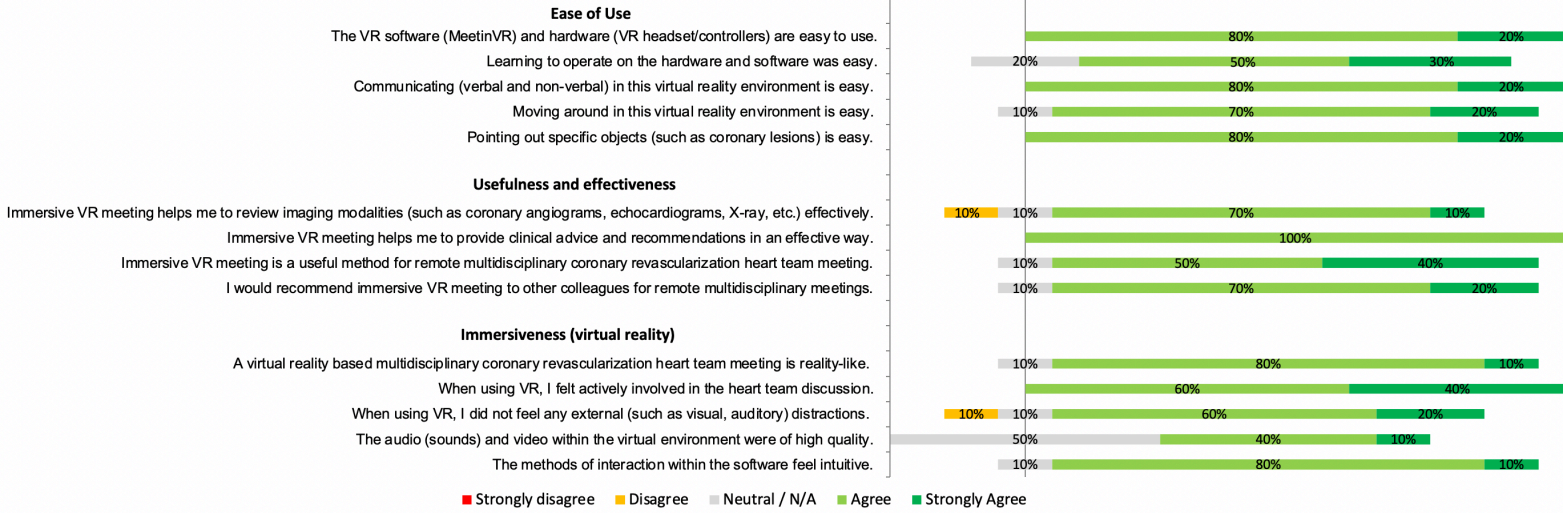
Questionnaire results on ease of use, usefulness and effectiveness, and immersiveness of virtual reality (VR) software and hardware for remote heart team meetings.

An overview on the results of the questionnaires is presented in figure 2. In general, VR-based meetings were rated as an easy-to-use, useful and effective method for remote heart team meetings. The participants were also asked to fill out advantages and disadvantages of VR-based meetings. These results are presented in online supplemental file S2. Some of the most common advantages were user-friendliness, safety, engagement, (especially during social distancing) and pointing out specific lesions in VR by all participants. Important (potential) disadvantages were the dependency on IT-infrastructure, the quality of the images and the dependency and comfort of wearing VR-hardware.

10.1136/bmjinnov-2021-000662.supp2 Supplementary data

### Alternative methods and future use

Ease-of-use, usefulness and effectiveness were rated to be better than tele/video conferencing by 90% of the users. Interestingly, when compared with physical meetings, 50% of the users rated VR-meetings to be similar and 20% rated VR-meetings to be much better. In addition, immersiveness was rated better than tele/video conferencing by 90% of the users. Refer to online supplemental figure 1 for a detailed overview of these results.

10.1136/bmjinnov-2021-000662.supp3 Supplementary data

Ninety per cent of the participants rated VR meetings to be a good method for future remote meetings and would like to work with this technology in the future and 80% of the users thought that in the future, they would even prefer working with this technology rather than tele/video conferencing. However, when compared with physical meetings, 50% did not prefer VR. See online supplemental figure 2 for a detailed overview.

10.1136/bmjinnov-2021-000662.supp4 Supplementary data

## Discussion

In this study we present the first examination of VR-based remote multidisciplinary heart team meetings to overcome social distancing challenges due to COVID-19. Remote meetings were organised in immersive VR using HMD and controllers and by providing a clinical case of a patient with coronary disease. We found that in general, the user experience was rated positive and that there was a positive attitude towards the use of VR as an alternative method for remote conferencing. An appreciated feature was found to be the possibility interact and to point out lesions directly with a virtual laser-pointer. This seemed to be an important shortcoming of 2D tele/video conferencing methods, where only one user can point out lesions (with a mouse arrow) when he/she is actually sharing his/her screen. Another advantage that was mentioned frequently was the engagement in the meeting. Due to all immersive features, the participants felt actively involved in the meetings and did not experience visual or auditory distractions from their surroundings. In addition, communication was mentioned to be intuitive and as good as a physical meeting, which underlines the advantages of immersive VR even more. Based on the questionnaire results and the experiences, it seems of the utmost importance that a VR device should be easy to use, light in weight, applicable to different types of software and preferably unwired. In addition, it should be possible to wear the headset when wearing glasses. For future clinical implementation, it is important to design a highly secured platform which is connected to the electronic health record, so a large set of patient data can be uploaded without any delay or inefficient anonymisation procedures. Moreover, a platform is needed that offers high security and compliance standards. For regular clinical implementation during a pandemic, hygienic measures should be taken into account as well. Even though most participants were excited using this novel VR technology, also some shortcomings and disadvantages were mentioned. For example, there seemed to be quite some room for improvement in terms of image quality. In some cases, the pixels on the shared monitor in the virtual meeting room were visible and thus the angiography images seemed to be a little less clear. However, most participants (80%) did not feel that this resulted in a less effective assessment of imaging modalities (figure 2).

With regard to the future, an interesting application of immersive technology and televirtuality would be the possibility of a real-time and remote evaluation of a patient through holographic telepresence and mixed reality technology.^14^ Finance is another important factor in considering structural clinical implementation and therefore a cost-effectiveness study would be desirable. A recent review on the use of telemedicine for multidisciplinary meetings demonstrated that some of the important advantages that telemedicine has to offer are decreased burdens of travel, a reduction of travel expenses and a reduction of overtime.^15^ Lack of acceptance associated costs of technology and suboptimal availability of an IT-infrastructure were identified as possible challenges and barriers for implementation of telemedicine for multidisciplinary meetings.

Besides enabling remote multidisciplinary meetings, VR technology has the potential to result in further advances in medicine and could be beneficial for both patients and physicians. Specifically, during these challenging times, alternative simulation and communication methods can be beneficial for physicians to cope with the current restriction rules due to COVID-19 but might also be a valid option for the future.^16 17^ More development, research and validation in technology could hopefully pave the way for a fully remote and immersive experience for the future of clinical medicine. Finally, we believe that future studies, comprising several cases and larger datasets that directly compare VR-based methods to other alternatives (eg, tele/video conferencing) are needed to draw conclusions.
