# Supplementary material for: Remote multidisciplinary heart team meetings in immersive virtual reality: a first experience during the COVID-19 pandemic
Source: BMJ Innov. 2021 Mar 5;7(2):311–5. doi: 10.1136/bmjinnov-2021-000662 (PMC7938471; doi:10.1136/bmjinnov-2021-000662)
Supplement: Supplementary data [file bmjinnov-2021-000662supp001.pdf]

Supplementary file S1

Immersive virtual reality coronary revascularization heart team meeting questionnaire

How often do you participate in coronary revascularization heart team meetings?

1. I never participate in coronary revascularization heart team meetings
2. I participate incidentally (less than once per month) in coronary revascularization heart team meetings
3. I participate sometimes (1-2 times / month) in coronary revascularization heart team meetings
4. I participate often (every week) in coronary revascularization heart team meetings

How many years of experience do you have in participating in coronary revascularization heart team meetings?

1. I have 0-1 years of experience in participating in coronary revascularization heart team meetings
2. I have 1-3 years of experience in participating in coronary revascularization heart team meetings.
3. I have 3-5 years of experience in participating in coronary revascularization heart team meetings.
4. I have >5 years of experience in participating in coronary revascularization heart team meetings.

How often do you use Virtual Reality hardware/software (e.g. virtual reality gaming, virtual reality simulations, virtual reality consoles, etc.)?

1. I have no experience (I <u>never</u> had VR experience until today)
2. I have basic experience (I have had some <u>incidental</u> VR experiences before, e.g. gaming, entertainment, etc.)
3. I am experienced (I use VR consoles and applications on a <u>regular basis</u>).
4. I am an expert (I <u>have a VR console</u> and applications myself).

Do you have any experience in immersive (virtual reality-based) remote meetings?

1. Yes
2. No

Ease of Use

1. The VR software (MeetinVR) and hardware (VR headset/controllers) are easy to use.

5<br>Strongly Agree	4<br>Agree	3<br>Neither Or N/A	2<br>Disagree	1<br>Strongly Disagree

2. Learning to operate on the hardware and software was easy.

5<br>Strongly Agree	4<br>Agree	3<br>Neither Or N/A	2<br>Disagree	1<br>Strongly Disagree

3. Communicating (verbal and non-verbal) in this virtual reality environment is easy.

5<br>Strongly Agree	4<br>Agree	3<br>Neither Or N/A	2<br>Disagree	1<br>Strongly Disagree

4. Moving around in this virtual reality environment is easy.

5<br>Strongly Agree	4<br>Agree	3<br>Neither Or N/A	2<br>Disagree	1<br>Strongly Disagree

5. Pointing out specific objects (such as coronary lesions) is easy.

5 Strongly Agree	4 Agree	3 Neither Or N/A	2 Disagree	1 Strongly Disagree

Usefulness and effectiveness

1. Immersive VR meeting helps me to review imaging modalities (such as coronary angiograms, echocardiograms, X-ray, etc.) effectively.

5 Strongly Agree	4 Agree	3 Neither Or N/A	2 Disagree	1 Strongly Disagree

2. Immersive VR meeting helps me to provide clinical advice and recommendations in an effective way.

5 Strongly Agree	4 Agree	3 Neither Or N/A	2 Disagree	1 Strongly Disagree

3. Immersive VR meeting is a useful method for remote multidisciplinary coronary revascularization heart team meeting.

5 Strongly Agree	4 Agree	3 Neither Or N/A	2 Disagree	1 Strongly Disagree

4. I would recommend immersive VR meeting to other colleagues for remote multidisciplinary meetings.

5<br>Strongly Agree	4<br>Agree	3<br>Neither Or N/A	2<br>Disagree	1<br>Strongly Disagree

Immersiveness (virtual reality)

1. A virtual reality based multidisciplinary coronary revascularization heart team meeting is reality-like.

5<br>Strongly Agree	4<br>Agree	3<br>Neither Or N/A	2<br>Disagree	1<br>Strongly Disagree

2. When using VR, I felt actively involved in the heart team discussion.

5<br>Strongly Agree	4<br>Agree	3<br>Neither Or N/A	2<br>Disagree	1<br>Strongly Disagree

3. When using VR, I did not feel any external (such as visual, auditory) distractions.

5<br>Strongly Agree	4<br>Agree	3<br>Neither Or N/A	2<br>Disagree	1<br>Strongly Disagree

4. The audio (sounds) and video within the virtual environment were of high quality.

5<br>Strongly Agree	4<br>Agree	3<br>Neither Or N/A	2<br>Disagree	1

				Strongly Disagree

5. The methods of interaction within the software feel intuitive.

5<br>Strongly Agree	4<br>Agree	3<br>Neither Or N/A	2<br>Disagree	1<br>Strongly Disagree

Alternative meeting methods

1. Regarding <u>ease-of-use</u>, **virtual reality** conferencing is a…….. way for organizing remote heart team meetings when compared to **tele/video** conferencing.

5<br>Much Better	4<br>Somewhat better	3<br>Similar	2<br>Somewhat worse	1<br>Much Worse

2. Regarding <u>usefulness and effectiveness</u>, **virtual reality** conferencing is a……… way for organizing remote multidisciplinary heart team meetings when compared to **tele/video** conferencing.

5<br>Much Better	4<br>Somewhat better	3<br>Similar	2<br>Somewhat worse	1<br>Much Worse

3. Regarding <u>usefulness and effectiveness</u>, **virtual reality** conferencing is a……… way for organizing remote multidisciplinary heart team meetings when compared to **physical meetings**.

5	4	3	2	1

Much Better	Somewhat better	Similar	Somewhat worse	Much Worse

4. Regarding <u>immersiveness (engagement in virtual environment)</u>, **virtual reality** conferencing is a……… way for organizing remote multidisciplinary heart team meetings when compared to **tele/video** conferencing.

5 Much Better	4 Somewhat better	3 Similar	2 Somewhat worse	1 Much Worse

Attitude towards (future) use

1. VR meetings are a good method for future remote coronary revascularization heart team meetings.

5<br>Strongly Agree	4<br>Agree	3<br>Neither Or N/A	2<br>Disagree	1<br>Strongly Disagree

2. I would like to work with this technology in the future.

5<br>Strongly Agree	4<br>Agree	3<br>Neither Or N/A	2<br>Disagree	1<br>Strongly Disagree

3. I enjoyed using VR for remote multidisciplinary meetings.

5<br>Strongly Agree	4<br>Agree	3<br>Neither Or N/A	2<br>Disagree	1<br>Strongly Disagree

4. In the future, I prefer using virtual reality conferencing methods over **tele/video** conferencing methods.

5<br>Strongly Agree	4<br>Agree	3<br>Neither Or N/A	2<br>Disagree	1<br>Strongly Disagree

5. In the future, I prefer using virtual reality conferencing methods over **physical** conferencing methods.

5<br>Strongly Agree	4<br>Agree	3<br>Neither Or N/A	2<br>Disagree	1<br>Strongly Disagree

Please provide advantages and disadvantages of immersive VR technology for organizing multidisciplinary meetings (please rank in order of importance *(start with the most important (dis)advantages)*

Advantages:

1. …….
2. …….
3. …….

Disadvantages:

1. …….
2. …….
3. …….

Did you feel that there were missing features in this virtual reality environment?

…..

Thank you for your participation
