# Supplementary material for: Remote multidisciplinary heart team meetings in immersive virtual reality: a first experience during the COVID-19 pandemic
Source: BMJ Innov. 2021 Mar 5;7(2):311–5. doi: 10.1136/bmjinnov-2021-000662 (PMC7938471; doi:10.1136/bmjinnov-2021-000662)
Supplement: Supplementary data [file bmjinnov-2021-000662supp002.pdf]

Supplementary file S2

Participant's opinion on advantages and disadvantages of remote multidisciplinary coronary revascularization heart team meetings in virtual reality.

Participant 1
Advantages:
1. Avoidance of distraction from surroundings.
2. Clear audiovisual content.
3. Attractive virtual meeting room.

Disadvantages:
1. Wearing glasses.
2. Sharpening the images for participants.
3. Costs?

Participant 2
Advantages:
1. No need to travel.
2. Decrease the risk of infection during COVID-19 pandemic.

Disadvantages:
1. Quality of the videos.

Participant 3
Advantages: none.
Disadvantages: none.

Participant 4
Advantages:
1. Could be of great help to organize "physical-like" meetings to meet colleagues in other/remote hospitals.

Disadvantages:
1. None.

Participant 5
Advantages:
1. Ease of use.
2. Good visualization of angiography/chest X-ray of patient.
3. Good communication.

Disadvantages:
1. Dependent upon internet.

Participant 6
Advantages:
1. Great method to engage in the heart team discussions (or other meetings) requiring participants interaction with the data showed (in this case angio and echo). This is not quite possible with video/teleconferences.
2. Best option for remote meetings during these pandemic times.
3. Good sound and user friendly.

Disadvantages:
1. It requires to be in possession of a VR equipment (somehow expensive?).

2. As an alternative to physical meetings during the pandemic it might mandate specific protocols for use in order to keep the risk of virus transference between users low.
3. Some people might not tolerate the virtual reality environment and might feel nausea or dizziness.

Participant 7

Advantages:

1. Distance is not a burden anymore.
2. Integration of images, also 3D/VR is possible.
3. It was OK when more people speak together.

Disadvantages:

1. Wearing VR glasses is awful compared to watch a screen!
2. Even more dependent on IT infrastructures and stability of networks.

Participant 8

Advantages:

1. Intuitive way of communication.
2. Pointing out lesions is doable in VR, but not in zoom/teams (video conferencing).
3. Fully immersive, less distraction from surroundings.

Disadvantages:

1. Quality of images (pixels can be seen).
2. Somewhat time-intensive to set up everything (in this proof of concept setup at least).
3. Delay in connection sometimes.

Participant 9

Advantages:

1. Direct contact with meeting participants.
2. Viewing images together with the possibility to interact.

Disadvantages:

1. The above may not necessarily require VR; online communication and sharing images can also be done with other methods.
2. Image quality of coronary angiogram was suboptimal.
3. We would need methods to view images in original quality. In fact, this is more important than the way of communication.

Participant 10

Advantages:

1. No travel time.
2. As good as physical meeting.
3. Use of pointer by all the participants.

Disadvantages:

1. No sharp images
2. Learning curve to handle tool.
