# Supplementary figures and images for: Remote multidisciplinary heart team meetings in immersive virtual reality: a first experience during the COVID-19 pandemic
Source: BMJ Innov. 2021 Mar 5;7(2):311–5. doi: 10.1136/bmjinnov-2021-000662 (PMC7938471; doi:10.1136/bmjinnov-2021-000662)

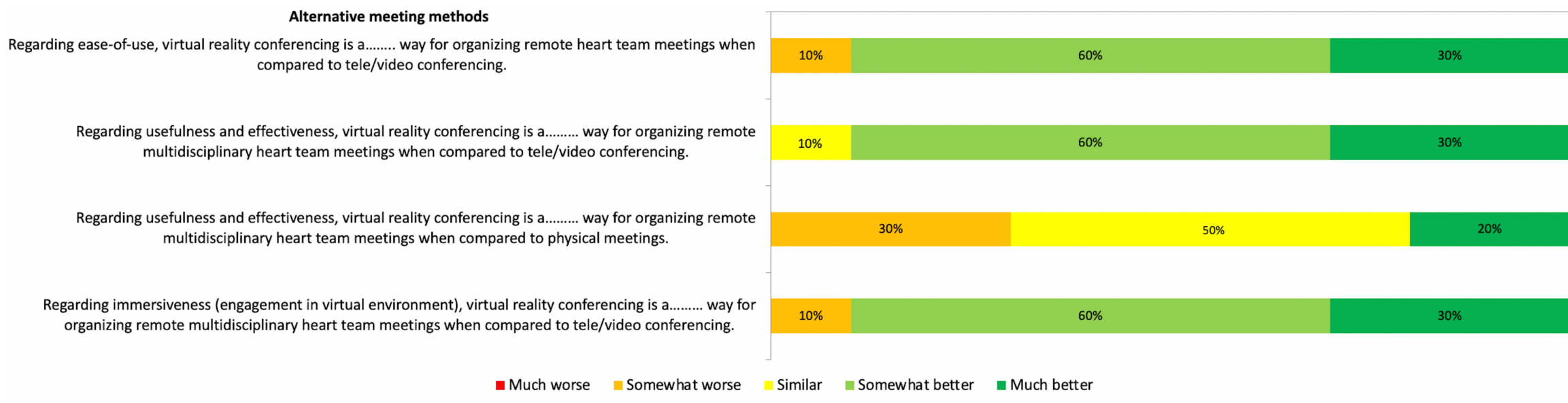

Supplement: Supplementary data [file bmjinnov-2021-000662supp003.pdf]

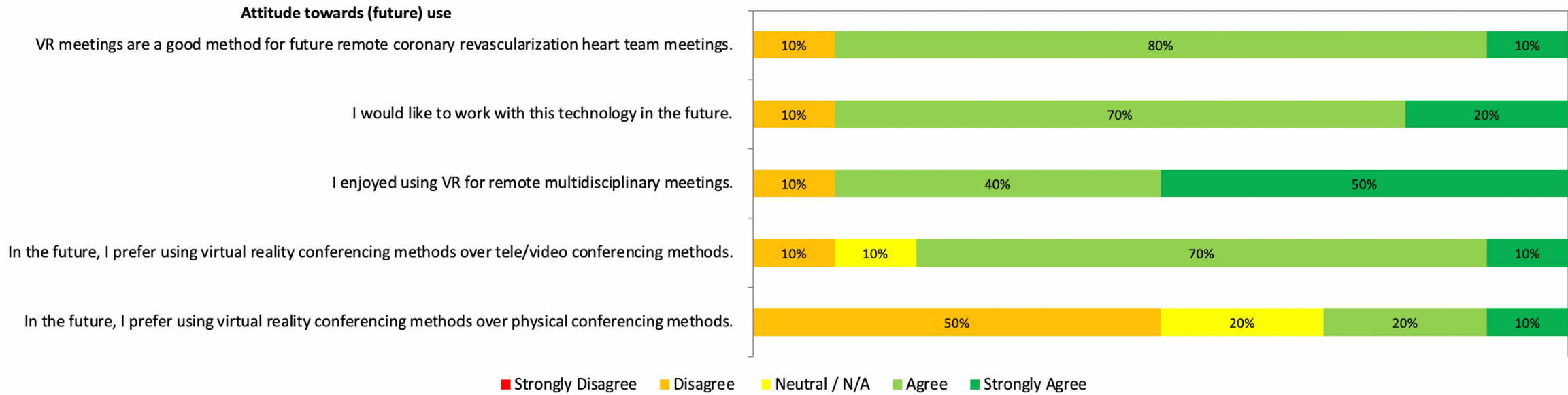

Supplement: Supplementary data [file bmjinnov-2021-000662supp004.pdf]
